# To what extent do callers follow the advice given by a non-emergency medical helpline (NHS 111): A retrospective cohort study

**DOI:** 10.1371/journal.pone.0267052

**Published:** 2022-04-21

**Authors:** Mable Angela Nakubulwa, Geva Greenfield, Elena Pizzo, Andreas Magusin, Ian Maconochie, Mitch Blair, Derek Bell, Azeem Majeed, Ganesh Sathyamoorthy, Thomas Woodcock

**Affiliations:** 1 Department of Primary Care and Public Health, School of Public Health, Imperial College London, London, United Kingdom; 2 Department of Applied Health Research, University College London, London, United Kingdom; 3 NHS North and East London Commissioning Support Unit, London, United Kingdom; 4 Department of Paediatric Emergency Medicine, Division of Medicine, St. Mary’s Hospital–Imperial College NHS Healthcare Trust, London, United Kingdom; University of Genoa, ITALY

## Abstract

National Health Service (NHS) 111 helpline was set up to improve access to urgent care in England, efficiency and cost-effectiveness of first-contact health services. Following trusted, authoritative advice is crucial for improved clinical outcomes. We examine patient and call-related characteristics associated with compliance with advice given in NHS 111 calls. The importance of health interactions that are not face-to-face has recently been highlighted by COVID-19 pandemic. In this retrospective cohort study, NHS 111 call records were linked to urgent and emergency care services data. We analysed data of 3,864,362 calls made between October 2013 and September 2017 relating to 1,964,726 callers across London. A multiple logistic regression was used to investigate associations between compliance with advice given and patient and call characteristics. Caller’s action is ‘compliant with advice given if first subsequent service interaction following contact with NHS 111 is consistent with advice given. We found that most calls were made by women (58%), adults aged 30–59 years (33%) and people in the white ethnic category (36%). The most common advice was for caller to contact their General Practitioner (GP) or other local services (18.2%) with varying times scales. Overall, callers followed advice given in 49% of calls. Compliance with triage advice was more likely in calls for children aged <16 years, women, those from Asian/Asian British ethnicity, and calls made out of hours. The highest compliance was among callers advised to self-care without the need to contact any other healthcare service. This is one of the largest studies to describe pathway adherence following telephone advice and associated clinical and demographic features. These results could inform attempts to improve caller compliance with advice given by NHS 111, and as the NHS moves to more hybrid way of working, the lessons from this study are key to the development of remote healthcare services going forward.

## Introduction

Medical telephone helplines have become available to patients in many western countries as the first point of contact [1, 2], usually for urgent care particularly during out-of-hours [2–4]. Such helplines provide 24/7 medical advice services, which enable callers to speak with a health professional about their medical concerns to assess clinical need, gain advice and reassurance, with onward referral to a range of other services as appropriate [5–7]. Helplines such as National Health Service (NHS) 111 were developed to effectively function as a triage system to direct callers to the right service and alleviate pressures on emergency services and unnecessary calls to 999 emergency ambulance service [8, 9].

With triage pathways based on evidence-based practice, one would expect that patients would follow the advice. However, if they are not reassured, they may seek care from other settings, such as emergency services, particularly during out-of-hours. This may have a negative impact on overcrowding Emergency Departments (EDs) with non-urgent cases. It is therefore important to investigate the extent to which callers follow triage advice and understand the factors associated with non-compliance.

In this paper, the caller’s action is defined to be ‘compliant’ with the advice given if the first subsequent service interaction in the flow is with a service that is consistent with the advice given. This definition aligns with previously published conceptualisation of triage compliance [10]. A meta-analysis in 2012 suggested that patient compliance with the advice given in telephone triage helplines was 62% [11]. In an Australian study with adults aged 45 years and older, there was 68.6% compliance with “Attend ED immediately” advice, 64.6% with “See a doctor” advice and 77.5% with “Self-care” advice, while self-referral to ED within 24h followed 7.0% of calls [10]. In another study, 66.5% of callers were compliant with advice to attend an ED, while 6.2% with low-urgency dispositions self-referred to the ED within 24 hours [12]. Callers that were compliant callers were significantly less likely to be triaged to the least urgent ED triage category compared to the general population [12]. The main reported reasons for non-compliance are having heard different advice, as well as callers’ intentions and health beliefs. For example, compliance was high if the triage advice matched the patient’s initial expectations for care [13].

### NHS 111

The emergency care system in England includes hospital EDs, hospital-based walk-in urgent care (WIC) centres, emergency ambulance, minor injury unit (MIUs), and General Practitioner in- and out-of-hours (GP OOH) services. The NHS 111 telephone service was established in 2010 to improve access to urgent care in England. NHS 111 was set up as a helpline for non-emergency matters, alongside 999 which is set up for emergency, life-threatening situations. Its purpose is to provide advice and to signpost people to the right level of service, whether a GP, a Walk-in Centre or an ED. The NHS provides guidance to the public about the appropriate services per the severity of their situations, with guidance when to call 111 or 999 (https://www.nhs.uk/nhs-services/urgent-and-emergency-care-services/when-to-call-999/). The service is operated 24/7 by non-clinical call handlers who triage calls to different services using clinical algorithms. Call handlers can also directly dispatch an emergency ambulance, and/or book appointments for callers to other telephone-based services.

Early evaluations of the NHS 111 revealed low awareness of the service [14], and no improvement in perceptions of urgent care or the health service [15]. In following years, the usage of NHS 111 has significantly increased [16].

Analysis of unadvised, non-urgent type 1 (considered “avoidable”) emergency department attendance after contact with the NHS 111 using national data showed that for every 20 calls where patients were not advised to attend ED, one resulted in and ED attendance within 24 hours [8]. Calls were less likely to result in an ED attendance when the patient received direct clinical input but more likely when the patient was a woman or children aged 0–4 years [8]. In a paediatric study analysing 11,279 calls to NHS 111 in Northwest London, 18.5% of callers were advised to attend an ED, and 63.8% of these attended within 10 hours. Callers who spoke to a GP during the NHS 111 call (rather than with a nurse) were less likely to attend an ED than other callers who did not speak with a GP [17].

The outcome measure in these studies was an ED attendance following an NHS 111 call. We aimed to explore a broader range of endpoints after and NHS 111 calls, including access to GPs, access to WIC centres, and ambulance dispatches. Referring patients to the correct pathway is essential to deliver safe and cost-effective care and understanding factors that increase patient compliance with the advice is crucial to provide the best care. It is therefore important to investigate the extent to which callers follow triage advice and understand the factors associated with non-compliance. The objective of this study was to investigate the extent to which callers comply with triage advice from the NHS helpline and explore factors associated with non-compliance following first interaction with 111 for a given complaint.

## Methods

### Data linkage

Datasets were linked and de-identified by Northeast London Commissioning Support Unit (NEL CSU). Firstly, we obtain time-ordered sequences of service interactions (i.e., NHS 111, GPOOH, WIC, EDs; MIUs, and ambulance services) for each patient. We grouped these sequences into sets of service interactions relating to distinct clinical complaints. Each of these sequences is referred to as a ‘patient flow’. Simply put, a ‘flow’ is an ordered series of service interactions that a patient has undertaken in response to a specific clinical complaint. By this definition, any given patient can have several ‘flows’ related to different conditions and involving various services and further calls to NHS 111. A flow can last hours, days, weeks, months, or years. Finally, to determine reasons for calling NHS 111, we used symptom groups, “READ codes”, and presenting complaint information, relating to NHS 111, GP OOH and ED data, respectively.

### Study design

This was an observational study, using linked data from the NHS 111 helpline and other urgent services.

We excluded records where any of the following important variables were missing: patient identity number, date-time stamp, unique reference code (for linking patient records from NHS 111 through to primary and/or secondary care services), triage advice, gender, age and ethnicity. We excluded flows containing no 111 calls; events within a flow that occurred before the first 111 call in that flow; and records with inconsistent or implausible patient sociodemographic information, including records where age recorded in different service records was inconsistent (differed by more than one year).

### Patients and callers

The study cohort comprised patients of all ages for whom at least one call was made to the London NHS 111 helpline between October 2013 and September 2017 (N = 1,964,726). The person making the call—the caller—was not always the patient (e.g., a parent calling about a child or a carer calling on behalf of the patient, where often the caller might be the decision-maker as well). Furthermore, data concerning the demographics of the caller were missing in over half of the call records and therefore were excluded from further analysis.

### Outcome variable—Compliance with advice

The outcome variable was compliance with the advice given in the NHS 111 call. Our focus was on compliance with the advice given on the first contact for a particular clinical complaint (I.e., our analysis was restricted to events following the first NHS 111 call within each flow). The caller’s action is defined to be ‘compliant’ with the advice given if the first subsequent service interaction in the flow is with a service that is consistent with the advice given. This definition aligns with the previous conceptualisation of triage compliance [10]. We developed a classification of whether each action taken by callers was consistent with each type of advice given. For example, if a caller was advised to attend ED but the next service interaction was a GP OOH, this flow would be considered non-compliant. Where the advice given was for the caller not to attend or contact another service, this was designated “self-care”, and the caller was deemed to comply with this advice if they did not subsequently interact with any service in relation to the same clinical complaint.

### Predictors

Patient sociodemographic characteristics include age, sex, and ethnicity, as recorded at first contact with NHS 111. Call features include the time of call, and NHS 111 triage advice.

### Statistical analysis

We examined distributions of NHS 111 patient characteristics, and urgent and emergency service usage following the first contact with NHS 111. Compliance with triage advice was described through a contingency table of the advice given against the action taken by the patient. Factors associated with compliance with triage advice were investigated using contingency tables, and a multiple logistic regression model [18] for compliance. The binary variable was compliance, predicted by gender; age group; ethnicity; triage advice; and time of the call.

For the regression analysis, flows whose first call to NHS 111 was missing triage disposition information were also excluded because it was not possible to determine whether they adhered to advice or not. It was impossible to determine the outcomes of calls for which the disposition was to attend other services such as other agencies, midwife or labour suites, social services. This is because these services were often not captured in subsequent data, meaning that it was not possible to determine if a caller had complied with triage advice. For that reason, calls with this disposition were excluded from the regression analysis. Results are reported as odds ratios with 95% CI. We conducted the analysis using the R statistical software (version 3.0.0 for Windows).

### Ethics statement

Research ethics approval was not required for this study, as it involved retrospective analysis of de-identified data, and the NHS Digital Confidentiality Advisory Group approved the use of the data for the purposes and stated aims of this study. Provider Caldicott Guardians approved the use of the data for the purposes of this study, including the linkage of de-identified data as outlined above.

## Results

The data linkage process identified 18,970,319 unique flows between October 2013 and September 2017. Of these, 3,703,094 flows contained at least one NHS111 call. Following exclusion of records with missing data, the final dataset had 3,579,786 flows relating to 1,964,726 patients.

### Patient characteristics

The majority of the calls were made for women, adults aged 30–59 years and for those of white ethnicity (Table 1).

**Table 1:** Socio-demographic characteristics of 1,964,726 patients at first contact with the NHS 111 service.

	Patients (n = 1,964,726)
**Sex**	
Women	1,130,984 (58%)
Men	818,742 (41%)
Unknown	15,000 (1%)
**Total**	**1,964,726 (100%)**
**Age Group**	
<12-months	127,759 (7%)
1–4	177,917 (9%)
5–15	165,952 (8%)
16–29	504,695 (26%)
30–59	647,058 (33%)
60–79	202,119 (10%)
>80	127,278 (6%)
Unknown	11,948 (1%)
**Total**	**1,964,726 (100%)**
**Ethnicity**	
Asian/Asian British	170,578 (9%)
Black/African/Caribbean/Black British	126,071 (6%)
Mixed/Multiple ethnic groups	63,442 (3%)
Other ethnic groups	40,352 (2%)
Unknown	848,959 (43%)
White	715,324 (36%)
**Total**	**1,964,726 (100%)**

### Reasons for calling NHS 111

Reasons for calling were grouped into high-level categories, since there were a large number of distinct symptoms and other reasons recorded. The ten most frequent reasons for calling NHS 111 are summarised in Table 2. These reasons accounted for 37% of the first calls to NHS 111 within each flow. For the remaining calls, the specified reason occurred less frequently, or the reason for calling was unknown.

**Table 2:** The most frequent reasons for calling NHS 111 within each of 3,579,786 ‘Flows’.

The ten most frequent reasons for calling NHS 111		
Symptoms	Total Flows (n = 3,579,786)	% Cumulative Frequency
Unclear urgent condition—ambulance ordered*	282,610 (8.0%)	8.0%
Vomiting, cough, hiccups, bringing up blood	162,738 (5.0%)	12.4%
Head, facial or neck injury	144,821 (4.0%)	16.5%
Health and social information	122,421 (3.0%)	20.0%
Dental Injury, bleeding, toothache or teething	118,662 (3.0%)	23.2%
Diarrhoea and / or vomiting, pregnant over 20 Weeks	106,980 (3.0%)	26.2%
Itch, skin problems	104,515 (3.0%)	29.1%
Genital area problems with or without foreign body	103,123 (3.0%)	32.0%
Chest or upper back Injury or pain	95,273 (3.0%)	34.7%
Pain and/or frequency passing urine	80,946 (2.0%)	37.0%
Other reasons (less frequently occurring reason groups)	1,224,420 (34%)	71.0%
Unknown	1,033,277 (29%)	100%

*The most frequent documented reason was ‘NHS Pathways in house Clinician’ (8%). According to Turner et al, this category represents ‘Unclear urgent condition—Ambulance ordered’ [9].

### Rates of compliance with triage advice

For 759,138 (33.2%) of the 3,579,786 flows included in this analysis, the advice given was documented, in the remaining 2,391,591 (66.8%) was missing. The most common advice given was to attend primary and community care (18.2%). The highest (83%) compliance was observed for calls where advice was self-care (Table 3). Further information on subsequent service interactions following the first NHS 111 call is presented in S1 Table in S1 File.

**Table 3:** The rates of compliance with triage advice for each of 3,579,786 ‘Flows’.

Advice given by NHS 111	Action taken by caller	Compliant	Frequency (%)
Ambulance Dispatches 123,627 (3.5%)	111	No	1,056 (1%)
	999 –ambulance dispatch	Yes	85,532 (69%)
	ED	No	15,750 (13%)
	GP OOH	No	8,696 (7%)
	UCC/MIUs/WIC	No	608 (0%)
	No service	No	11,985 (10%)
	**Total**		**123,627 (100%)**
Self-care 257,949 (7.2%)	111	No	10,121 (4%)
	999	No	273 (0%)
	ED	No	12,477 (5%)
	GP OOH	No	18,077 (7%)
	UCC/MIUs/WIC	No	3,853 (1%)
	No service	Yes	213,148 (83%)
	**Total**		**257,949 (100%)**
Advised to Attend ED 137,897 (3.9%)	111	No	806 (1%)
	999	No	704 (1%)
	ED	Yes	58,431 (42%)
	GP OOH	No	20,700 (15%)
	UCC/MIUs/WIC	No	9,862 (7%)
	No service	No	47,394 (34%)
	**Total**		**137,897 (100%)**
*Advised to Attend Other Service 14,279 (0.4%)	111	No	378 (3%)
	999	No	23 (0%)
	ED	No	743 (5%)
	GP OOH	No	6,911 (48%)
	UCC/MIUs/WIC	No	56 (0%)
	No service	No	6,168 (43%)
	**Total**		**14,279 (100%)**
Advised to Attend Primary and Community Care 654,443 (18.2%)	111	No	10,441 (2%)
	999	No	687 (0%)
	ED	No	46,600 (7%)
	GP OOH	Yes	227,339 (35%)
	UCC/MIUs/WIC	No	14,775 (2%)
	No service	No	354,601 (54%)
	**Total**		**654,443 (100%)**
Missing 2,391,591 (66.8%)	111	**-**	62,583 (2.6%)
	999	**-**	134,995 (5.6%)
	ED	**-**	286,511 (12%)
	GP OOH	**-**	521,870 (21.8%)
	UCC/MIUs/WIC	**-**	80,691 (3.4%)
	No service	**-**	1,304,941 (54.6%)
	**Total**	**-**	**2,391,591 (100%)**
Total 3,579,786 (100%)			

Column ‘Compliant’ represents the study outcome, and specifies our classification of compliance according to the first service interaction following the first call to NHS 111, within each flow.

*Advised to Attend Other Service: not possible to assess subsequent services as these are not captured sufficiently.

UCC = Urgent Care Centre; and MIUs = Minor Injury Units; WIC = Walk-in Centre. The unit of analysis is flows. “No service” here indicates that no further service interaction was recorded as part of the same flow.

### Factors associated with compliance with triage advice

The following results include only the 759,138 flows with documented triage advice. Overall, in 49% of calls, callers complied with triage advice. Compliance was highest in calls for children under the age of 5 (55%) and those aged 5–15 (52%). Compliance was almost identical among calls for men (50%) vs women (49%). Compliance was highest among those of Asian/Asian British (52%), and Mixed/Multiple ethnic groups (50%).

A higher percentage (52%) of those calling out of hours compiled with triage advice, compared with those calling in hours (42%). Compliance was highest for callers advised they did not need to attend another service (81%), and those for whom an ambulance dispatch was advised (67%). Compliance was lower among those advised to attend ED (43%) and Primary and Community Care (35%).

The results in Table 4 reveal the biggest impact on compliance was the ‘advice given’. The differences in the proportion of callers that complied vs. those that did not, for the remaining variables were small.

**Table 4:** Univariate analysis of patient and call characteristics associated with compliance with NHS 111 triage advice: 759,138 flows with documented triage advice.

	Compliance n = 371,894 (49%)	Non-compliance n = 387,244 (51%)	Overall n = 759,138 (100%)	*P*
**Age group**				
<12-months	45,002 (55%)	36,225 (45%)	81,227	
1–4	42,188 (55%)	33836 (45%)	76,024	p < 0.001
5–15	26,945 (52%)	25,310 (48%)	52,255	
16–29	92,306 (46%)	109,455 (54%)	201,761	
30–59	101,326 (47%)	112,940 (53%)	214,266	
60–79	38,289 (49%)	39,747(51%)	78,036	
Elderly >80	25,838 (46%)	29,731(54%)	55,569	
**Sex**				
F	223,485 (49%)	236,596 (51%)	460,081	p < 0.001
M	148,409 (50%)	150,648 (50%)	299,057	
**Ethnicity**				
Asian/Asian British	61,024 (52%)	56,500 48%)	117,524	
African/Caribbean/Black British	42,130 (48%)	45,535 (52%)	87,665	
Mixed/Multiple ethnic groups	25,360 (50%)	24,862 (50%)	50,222	p < 0.001
Other ethnic group	10,694 (48%)	11,710 (52%)	22,404	
White	232,686 (48%)	248,637 (52%)	481,323	
**Time of call**				
Out of hours	278,446 (52%)	260,628(48%)	539,074	p < 0.001
In-hours	93,448(42%)	126,616 (58%)	220,064	
**Advice given**				
Ambulance	53,783 (67%)	25,941 (33%)	79,724	
Self-care	126,791 (81%)	29,455 (19%)	156,246	p < 0.001
Attend A&E	38,568 (43%)	50,637 (57%)	89,205	
Primary and Community Care	152,752 (35%)	281,211 (65%)	433,963	

*P-values are chi-square test results. The time of call period included calls made during various times. After-Hours is defined as NHS’s out-of-hours capturing periods call activity between 6.30 pm to 8.00 am during weekdays and all day on weekends and bank holidays.

As in the univariate analysis above, results of multiple logistic regression analysis (Table 5) show that calls for younger children were most likely to be compliant, with the age group most likely to comply being for children aged < 12 months. Calls on behalf of children aged ≤ 4 years were only slightly less likely to comply (OR 0.90, 95% CI 0.88–0.92). The age group least likely to comply with triage advice was >80 (OR 0.67, 95% CI 0.65 to 0.68). Whilst compliance was only slightly lower in men compared with women (OR 0.95, 95% CI 0.94 to 0.96), this was nevertheless statistically significant. This small difference was not apparent in the univariate analysis and may be related to interactions between sex and other variables. As in the univariate analysis, compliance was highest among calls for those of Asian/Asian British ethnicity, with statistically significant differences in compliance between this group and other ethnicities (p < 0.001). Compliance with triage advice was significantly lower for calls made in-hours compared with out of hours (OR 0.59, 95% CI 0.58 to 0.59), consistent with the univariate results. Compared with callers where triage advice was ambulance dispatch, those advised to self-care were nearly twice as likely to comply with triage advice (OR 1.98, 95% CI 1.94 to 2.02). In contrast, those advised to attend ED and primary and community care’ were significantly less likely to comply with triage advice (OR 0.35, 95% CI 0.34 to 0.36; OR 0.24, 95% CI 0.24 to 0.24, respectively).

**Table 5:** Logistic regression model results for the association between patient and flow characteristics and compliance with triage advice (n = 759,138 flows with documented triage advice).

	OR (95% CI)	*P*
**Age**		
< 12 months*	1	
1–4	0.90 (0.88–0.92)	p < 0.001
5–15	0.81 (0.79–0.83)	p < 0.001
16–29	0.70 (0.69–0.71)	p < 0.001
30–59	0.72 (0.71–0.73)	p < 0.001
60–79	0.75 (0.73–0.76)	p < 0.001
>80	0.67 (0.65–0.68)	p < 0.001
**Sex**		
Women*	1	
Men	0.95 (0.94–0.96)	p < 0.001
**Ethnicity**		
Asian/Asian British*	1	
Black/African/Caribbean/Black British	0.84 (0.82–0.86)	p < 0.001
Mixed/Multiple ethnic groups	0.85 (0.83–0.87)	p < 0.001
Other ethnic groups	0.81 (0.78–0.83)	p < 0.001
White	0.86 (0.85–0.87)	p < 0.001
**Time of call**		
Out of hours*	1	
In-hours	0.59 (0.58–0.59)	p < 0.001
**Advice given**		
Ambulance*	1	
Self-care	1.98 (1.94–2.02)	p < 0.001
Attend A&E	0.35 (0.34–0.36)	p < 0.001
Attend Primary and Community Care	0.24 (0.24–0.24)	p < 0.001

*Reference categories for each variable.

## Discussion

### Summary of main findings

Overall, 49% of callers complied with triage advice. Patient demographic variables had a small but statistically significant effect on compliance with triage advice. Compliance was more likely in calls for children aged <16 years; women; and those of Asian/Asian British ethnicity. Call characteristics had a larger effect on compliance, with higher compliance (52%) among those calling out of hours compared with those calling in hours (42%). The advice given had the biggest effect on compliance, with 81% of those advised not to seek further care complying with this advice, compared with only 35% and 42% in those advised to attend primary care and ED respectively.

### How do these findings correspond with previous studies?

Whilst observed compliance reported in this study is lower than figures previously reported, it is important to highlight that there is significant variation in reported compliance rates, as well as differences in study designs and definitions of compliance. Some researchers found overall self-reported compliance of over 80% in a postal survey of NHS 111 patients [5]. A systematic review on the appropriateness of, and compliance with, telephone triage services reported compliance rates to range from 56% to 98% (median 77%) [19]. Many studies included in this review were based on self-reported data, and such studies overall reported higher compliance than those based on service interaction or billing data.

The higher compliance among calls on behalf of younger children seen in this study aligns with [12] who observed similar patterns in compliance to attend ED for children vs adults (defined as ≥ 17 years). Similarly, our finding that compliance is significantly more likely where the advice was ambulance referral or self-care is consistent with previous studies [2, 11]. A systematic review similarly found that the level of compliance with advice to attend primary care was lower than that for both emergency or urgent care and self-care [19].

Our finding of low compliance with advice to attend ED is unusual, with previous studies showing comparable compliance with advice to attend ED and to self-care [2, 19]. Of those who did not comply with advice to attend ED, most did not attend any other service, although some did contact GP out-of-hours services. The wide variety of health systems and telephone helplines covered by the existing literature means that comparisons of compliance results between studies should be interpreted with caution. For instance, studies such as the US study by [20] or [12] in Australia often have a system where after-hours triaging is done by nurses. Also, some telephone helpline services are not promoted as widely as the NHS 111 service is in England and are not as widely used by the public [12]. Furthermore, previous studies on compliance with telephone advice have often been assessed using follow-up self-report surveys or phone interviews. Although these study designs have been shown to identify patterns and reasons for non-compliance to triage advice, they are subject to bias through self-reporting [13] as well as small sample sizes [5, 13]. Such studies are also usually retrospective, asking callers to recall previous usage of telephone triage services.

### Policy implications

Telephone triage and advice services have become an acceptable component of delivering healthcare worldwide [10], with their increasing prominence driven by the high demand for urgent and emergency care services. These services have transformed patients’ access to healthcare [11], with large numbers of users and high levels of patient satisfaction [5]. Compliance with triage advice is an important outcome of telephone triage services, both in terms of ensuring patients receive the health care that they need and because it helps demonstrate the effectiveness of telephone helplines [11]. Compliance could be improved by providing timely, accessible, reassuring and compassionate advice., incorporating the patient’s wishes as much as possible. The NHS 111 is a step forward towards achieving this goal, as the information on the current concern is stored, and they do not need “to tell the whole story again” if they call again. Poor compliance with triage advice might lead to inefficient resource use, and impaired clinical outcomes. Improving compliance with advice could otherwise streamline and speed up the response to healthcare needs. For example, the closest ED to the patient’s home can be notified by NHS 111 about the patient’s referral to the ED and imminent arrival. This could shorten assessment and triage time and allow speedier response in urgent cases.

It is clear from the results of this study that the factors affecting compliance most strongly are call-related factors, the time of the call and the advice given, rather than the patient demographics. This has implications in terms of prioritising work to improve the effectiveness and efficiency of urgent and emergency care systems. First, the relatively low levels of compliance among callers advised to attend ED indicate the importance of understanding why callers do or do not follow this specific advice. Such an understanding could inform improvements in the algorithms used by telephone services in assessing risk to patients and what level of subsequent care is needed, as well as improving communication where necessary. For example, if it is found that callers with certain presenting complaints are advised to attend ED but largely do not do so, this could lead to adjustments in the algorithm used to recommend ED attendance, so that people are not unnecessarily directed to attend ED. There are important safety considerations in making such changes however, since it may not be possible to distinguish those at low and high risk fully through a telephone call, and for some presentations, recommending attendance at ED may still be the only safe option. Finally, compliance with 999 (reported as 69% in Table 3) may appear lower than expected. However, it is important to note that a 999 request may be downgraded to a slower response, suitable to the nature of the clinical features of the call.

We highlight the need for effective and patient-centred communication skills in a telephone consultation context, including active listening and active advising skills and structuring the conversation. Studies have found higher compliance when callers feel they can trust the advice given. For instance, 94.0% of patients reported they followed the advice given to them by GPs and for 86.8% of patients their reported actions following consultations matched the recommended advice documented by GPs in the Healthdirect database [21]. The same study concluded that improving patient satisfaction with the service and understanding of advice given can lead to an increased compliance rate [21].

These issues of trust and communication highlight that it is neither realistic nor desirable for callers to automatically “comply” with triage advice, but rather that they have preferences and choices that should be considered and respected. This aligns with the view of the person-centred approach, and patient choice. An Australian study investigating compliance with telephone helpline advice to attend an ED following a call [12] found that callers whose prior intention was to attend an ED were more likely to be compliant with dispositions to attend ED immediately, compared to callers with other prior intentions. This highlights the importance of considering the call advice in context. Furthermore, there are a wide variety of potential explanations for non-compliance. Studies investigating compliance with triage advice report that the most common reasons for non-compliance were recall problems, whereby patients reported hearing a different triage disposition than what was recorded, changing symptom severity (either increased or decreased), or because patients wanted a second opinion or additional advice from another healthcare practitioner [2, 6, 10, 13, 22]. This points to potential avenues for improved care, including interventions that could remind callers of the advice given after the call, such as what to do if their symptoms change, and where they might seek additional advice; and introducing an option for a video call to NHS111 instead of a phone call, to add a more personal communication aspect.

### Strengths and limitations

This study represents an analysis of a large, linked dataset covering 4.5 years of data, providing an in-depth overview of patient pathways following the first call to NHS 111 for a particular clinical complaint. This is the first comprehensive study using linked data to understand how patients use other urgent care services following contact with NHS 111. Using linked records of service use for those contacting telephone services offers a more understanding of ‘actual’ trends and patterns of compliance, compared with self-reported data. Other studies have been limited to a single endpoint service, such as ED, or have focussed on a specific age group rather than looking at service use across a population [10, 23, 24].

This study used the ‘flow’ method to separate service interactions with the same patient for different clinical symptoms or complaints. This method has the advantage that compliance with advice is measured in relation to the same symptom or complaint that the advice was given for. This is especially important in cases where a patient has multiple long-term conditions any of which may result in need of healthcare services.

As in many studies investigating administrative, routinely collected healthcare data, this data was affected by missing information [2, 3]. This included triage advice and the reason for calling. It is not clear why triage advice was missing for a significant proportion of calls. This is something that telephone helpline services should consider addressing in future, as improved completeness of this important field would strengthen future research into compliance. Data were not available on the actual and perceived severity of the clinical complaint, which could influence the likelihood that callers will follow advice given. There were also examples of patient socio-demographic information that was inconsistent across linked datasets, although this issue was not widespread. The “relationship to the caller” field was not well populated. For this reason, we were not able to analyse differences in compliance between callers calling on behalf of themselves, and those calling on behalf of someone else. Also, it was not possible to determine from the available data how WICC, UCC, and MIU centres were operated; for example, whether they were GP-led or nurse-led. Hence, caution should be exercised when interpreting the caller’s intention—going to a UCC might be a sensible action for a patient that has been advised to see a GP. For most calls it was also not possible to establish from the available data whether patients’ symptoms may have improved or worsened following the call; this may have impacted on their compliance with advice given. The data did not contain information on the nature of self-care administered by callers, or of care administered by others such as carers, social care providers, or private health providers. The self-care category may therefore include callers receiving such support.

The data for this study only included deaths occurring in hospital, and this analysis did not distinguish between patients who did or did not die within the study period. This is important when considering the impact of compliance on service use, to ensure all relevant service interactions were included.

### Future research

This study demonstrates the impact on compliance of caller demographics, call timing, and advice given. However, the completeness of data relating to the reason for calling and the clinical nature of the patient’s complaint was not sufficiently high to draw meaningful conclusions on the relationship between these factors and compliance, and further research is needed to understand this relationship. This would be facilitated by improved data completeness. Better data completeness would also facilitate future research exploring death and urgent and emergency care use as competing risks. The relatively low proportion of callers following advice to attend ED warrants further study, to understand whether there are factors within this particular subgroup that are associated with compliance. This may facilitate interventions targeted at improving advice and care for these groups. Qualitative research focussing on reasons why callers do not follow advice given, especially among the groups identified with lower compliance, may also aid improvement in this area.

## Conclusions

On average half of London’s callers to the NHS 111 helpline during the study period complied with the advice given in terms of healthcare services that they subsequently interacted with. This represents lower compliance than that is often reported in the literature. Compliance with the advice given by the helpline varies by caller demographics and by whether the call is made within working hours but varies most by the type of advice given. Where self-care or ambulance was advised, compliance with triage advice was relatively high. A record of ED attendance was only documented for 43% of callers advised to attend ED by the NHS 111 helpline. This may be an indication that the NHS 111 service over-estimates the urgency of some patients, potentially resulting in non-compliance or overuse of urgent care services. This suggests the potential for improvement in the accuracy of triage advice.

## Supporting information

S1 File (DOCX)

## References

[1] Tran DT, Gibson A, Randall D, et al. Compliance with telephone triage advice among adults aged 45 years and older: an Australian data linkage study. *BMC Health Serv Res* 2017; 17: 512. doi: 10.1186/s12913-017-2458-y 28764695 PMC5539620

[2] Kempe A, Bunik M, Ellis J, et al. How Safe Is Triage by an After-Hours Telephone Call Center? *PEDIATRICS* 2006; 118: 457–463. doi: 10.1542/peds.2005-3073 16882795

[3] Kempe A, Luberti AA, Hertz AR, et al. Delivery of Pediatric After-Hours Care by Call Centers: A Multicenter Study of Parental Perceptions and Compliance. *PEDIATRICS* 2001; 108: e111–e111. doi: 10.1542/peds.108.6.e111 11731638

[4] Belman S, Chandramouli V, Schmitt BD, et al. An Assessment of Pediatric After-hours Telephone Care: A 1-Year Experience. *Arch Pediatr Adolesc Med*; 159. Epub ahead of print 1 February 2005. doi: 10.1001/archpedi.159.2.145 15699308

[5] O’Cathain A, Knowles E, Turner J, et al. Acceptability of NHS 111 the telephone service for urgent health care: cross sectional postal survey of users’ views. *Family Practice* 2014; 31: 193–200. doi: 10.1093/fampra/cmt078 24334420 PMC3969523

[6] Mckew M. NHS 111: help or hindrance for emergency care?: Call handlers said to be directing too many people with ‘laughable’ conditions to front-line services. *Emergency Nurse* 2017; 24: 8–9. doi: 10.7748/en.24.10.8.s8 28279100

[7] Lewis J, Stone T, Simpson R, et al. Patient compliance with NHS 111 advice: Analysis of adult call and ED attendance data 2013–2017. *PLOS ONE* 2021; 16: e0251362. doi: 10.1371/journal.pone.0251362 33970946 PMC8109810

[8] Egan M, Murar F, Lawrence J, et al. Identifying the predictors of avoidable emergency department attendance after contact with the NHS 111 phone service: analysis of 16.6 million calls to 111 in England in 2015–2017. *BMJ Open* 2020; 10: e032043. doi: 10.1136/bmjopen-2019-032043 32152158 PMC7066618

[9] Turner J, O’Cathain A, Knowles E, et al. Impact of the urgent care telephone service NHS 111 pilot sites: a controlled before and after study. *BMJ Open* 2013; 3: e003451. doi: 10.1136/bmjopen-2013-003451 24231457 PMC3831104

[10] Tran DT, Gibson A, Randall D, et al. Compliance with telephone triage advice among adults aged 45 years and older: An Australian data linkage study. *BMC Health Services Research*; 17. Epub ahead of print 1 August 2017. doi: 10.1186/s12913-017-2458-y 28764695 PMC5539620

[11] Purc-Stephenson RJ, Thrasher C. Patient compliance with telephone triage recommendations: A meta-analytic review. *Patient Education and Counseling*. Epub ahead of print 2012. doi: 10.1016/j.pec.2011.08.019 22001679

[12] Gibson A, Randall D, Tran DT, et al. Emergency Department Attendance after Telephone Triage: A Population-Based Data Linkage Study. *Health Serv Res* 2018; 53: 1137–1162. doi: 10.1111/1475-6773.12692 28369871 PMC5867179

[13] Niemann S, Meer A, Simonin C, et al. Medical telephone triage and patient behaviour: How do they compare? *Swiss Med Wkly* 2004; 134: 126–131. doi: 2004/09/smw-10276 15106022 10.4414/smw.2004.10276

[14] Knowles E, O’Cathain A, Turner J, et al. Awareness and use of a new urgent care telephone service, NHS 111: cross-sectional population survey. *J Health Serv Res Policy* 2014; 19: 224–230. doi: 10.1177/1355819614535571 24819379

[15] Knowles E, O’Cathain A, Turner J, et al. Effect of a national urgent care telephone triage service on population perceptions of urgent care provision: controlled before and after study. *BMJ Open* 2016; 6: e011846. doi: 10.1136/bmjopen-2016-011846 27742622 PMC5073559

[16] Turner J, Knowles E, Simpson R, et al. Impact of NHS 111 Online on the NHS 111 telephone service and urgent care system: a mixed-methods study. *Health Services and Delivery Research* 2021; 9: 1–148. doi: 10.3310/hsdr09210 34780129

[17] Robinson C, Wolters A, Steventon A, et al. Predictors of emergency department attendance following NHS 111 calls for children and young people: analysis of linked data. *Clin Med (Lond)* 2017; 17: s13. doi: 10.7861/clinmedicine.17-3-s13 30958775 PMC6334155

[18] McCulloch P, Nelder JA. *Generalized Linear Models*. 2nd Edition. London: Chapman and Hall, 1990.

[19] Blank L, Coster J, O’Cathain A, et al. The appropriateness of, and compliance with, telephone triage decisions: a systematic review and narrative synthesis: *Telephone triage*. *Journal of Advanced Nursing* 2012; 68: 2610–2621. doi: 10.1111/j.1365-2648.2012.06052.x 22676805

[20] Lee TJ, Baraff LJ, Wall SP, et al. Parental Compliance with After Hours Telephone Triage Advice: Nurse Advice Service versus On-Call Pediatricians. *Clin Pediatr (Phila)* 2003; 42: 613–619. doi: 10.1177/000992280304200707 14552520

[21] Li L, Georgiou A, Xiong J, et al. Healthdirect’s After Hours GP Helpline—A Survey of Patient Satisfaction with the Service and Compliance with Advice. *Stud Health Technol Inform* 2016; 227: 87–92. 27440294

[22] Dale J, Crouch R, Patel A, et al. Patients telephoning A&E for advice: a comparison of expectations and outcomes. *Emergency Medicine Journal* 1997; 14: 21–23. 10.1136/emj.14.1.21 PMC1342837 9023617

[23] Staub GM, von Overbeck J, Blozik E. Teleconsultation in children with abdominal pain: a comparison of physician triage recommendations and an established paediatric telephone triage protocol. *BMC Med Inform Decis Mak* 2013; 13: 110. doi: 10.1186/1472-6947-13-110 24079719 PMC3849753

[24] Stewart B, Fairhurst R, Markland J, et al. Review of calls to NHS Direct related to attendance in the paediatric emergency department. *Emergency Medicine Journal* 2006; 23: 911–914. doi: 10.1136/emj.2006.039339 17130596 PMC2564250

