# Supplementary material for: To what extent do callers follow the advice given by a non-emergency medical helpline (NHS 111): A retrospective cohort study
Source: PLoS One. 2022 Apr 21;17(4):e0267052. doi: 10.1371/journal.pone.0267052 (PMC9022858; doi:10.1371/journal.pone.0267052)
Supplement: S1 File — (DOCX) [file pone.0267052.s001.docx]

**Supplementary Material**

**Supplementary S1: Further information on subsequent service interactions following the first NHS 111 call**

The number of contacts with services following for the first calls to NHS 111 within each flow Table S1). Here we consider the number of contacts with services, for each flow (n = 3,579,786) following initial contact with NHS 111. Flows comprising a single interaction (I.e., just an NHS 111 call and no further contact with urgent and emergency care services) are defined as having one interaction in their pathways. Excluding such Flows (54%), the majority of the flows (30%) had one other interaction with a U&EC service in their pathway. The number of NHS 111 calls within each flow: Of the 3,579,786 flows starting with an NHS 111 call, the majority (3,379,145, 94%) did not contain any further calls to NHS 111. 5% of the flows, were recorded as having one other call to NHS 111.

**Table S1: Characteristics of the first calls to NHS 111 within each ‘Flow’**

| **Number of contacts with services following contact with NHS 111 per flow** | | |
| --- | --- | --- |
| **Number of flows (n = 3,579,786)** | **Service events within each flow** | **% Cumulative Frequency** |
| 1,938,237 | 1 (54%) | 54.3% |
| 1,082,258 | 2 (30%) | 84.7% |
| 404,595 | 3 (11%) | 96.0% |
| 98,495 | 4 (3%) | 98.8% |
| 31,210 | 5 (1%) | 99.7% |
| 24,991 | > = 6 (1%) | 100% |
